# Gradient Structure Design and Welding-Hammering Hybrid Remanufacturing Process of Continuous Casting Rollers

**DOI:** 10.3390/ma15238588

**Published:** 2022-12-01

**Authors:** Jiansheng Zhang, Guiqian Xiao, Jie Peng, Yingyan Yu, Jie Zhou

**Affiliations:** 1 Chongqing Key Laboratory of Advanced Mold Intelligent Manufacturing, College of Materials Science and Engineering, Chongqing University, Chongqing 400044, China; 2 Chongqing Jiepin Technology Co., Ltd., Chongqing 400000, China

**Keywords:** welding-hammering hybrid, roller, failure mode, microscopic analysis

## Abstract

To improve the service life and reduce the repair cost of continuous casting rollers, a new welding-hammering hybrid remanufacturing process in which the roller was designed with a gradient structure was proposed, and corresponding equipment was developed. First, the failure modes and their causes for a continuous casting roller were analyzed by numerical simulation. The cyclic tension–compression shear stress, cyclic tension–compression normal stress, thermal cycle, and highly corrosive environment caused fatigue cracking and overall peeling of the roller surface. Second, the gradient structure composed of a base layer, transition layer, and strengthened layer of a continuous casting roller was designed, and materials for each layer were selected according to their different service conditions. Third, novel equipment for continuous welding-hammering composite remanufacturing was developed, and the optimized process parameters were obtained through welding experiments. Finally, an application test was carried out; the microscopic analysis showed that refined grains, fewer welding defects, and better surface toughness were obtained. Compared with traditional remanufacturing processes without hammering, the welding-hammering hybrid process achieved a forged structure instead of as-cast structure, which significantly improved the service life of the continuous casting roller by about 100%.

## 1. Introduction

The service life of continuous casting rollers is short under severe working conditions such as thermal-fatigue cycles, high-temperature oxidation, and mechanical abrasion [1,2], which raises production costs in the steel industry. To improve the service life and reduce the repair cost of continuous casting rollers, their remanufacturing process has been widely studied. The deposition of overlay coatings, by welding [3,4,5] or laser melting techniques [6], is frequently employed in industry, either during repair or in the manufacture of new components [7]. Chen et al. [8] used selective laser melting technology to deposit W-Ni-Fe-Co compound powders on rollers to explore solutions that enable fabrication of complex W composite parts by additive manufacturing. The results showed that the W-Ni-Fe-Co composite material could be consolidated on the roller surface and the wear resistance of the roller could be greatly improved. Koodziejczak et al. [9] used laser cladding to deposit Ni-Cr and Re layers, which dramatically improved the corrosion and oxide resistance of deposited coatings at high operating temperatures. Kołodziejczak et al. [10] used cold metal transfer to deposit four protective coating materials (Inconel 718, Inconel 625, Alloy 33, and Stellite 6) on 16Mo3 steel tubes. The hardness and wear resistance were tested. The results showed that the Stellite 6 layer was the hardest, at about 500 HV0.2. Other materials such as Inconel 625, Inconel 718, and Alloy 33 represented a clad zone hardness of about 250 HV0.2. The Stellite 6 layer had the lowest wear resistance in the dry sand/rubber wheel test, and the Stellite 6 layer had the highest wear resistance in the erosive blasting test. A numerical procedure for the hardfacing process based on a thermal cycle curve method was established by Tian et al. [11] to research the temperature and residual stress fields in the hardfacing remanufacturing for a large-scale grinding roller with damage. The numerical simulation results showed that with an increase in surface layers, the heat-affected zone of the grinding roller gradually expands, and the maximum tensile stress always appears at the position near the weld toe, which is prone to fatigue failure or interface peeling due to stress concentration. Deng et al. [12] used plasma transferred arc welding (PTAW) and oxyacetylene arc welding (OAW) to deposit a Co-based alloy layer on the surface of heat-resistant steel. The results showed that the fatigue strength of OAW specimens at room temperature was lower than that of PTAW specimens, which may be due to the higher number of carbides in OAW coatings. Amushahi et al. [13] studied the influence of arc spraying and gas metal arc welding technology on wear resistance of boride-rich coatings. The results showed that boride coatings obtained by welding had better adhesion to the steel substrate and better resistance to spalling. The effect of welding procedural variations in the cracking sensitivity and interface performance between substrate and deposited layers was studied by Chatterjee et al. [14]. Kesavan et al. [15] studied the effects of aging treatment on coating microstructure, wear and corrosion properties. They pointed out that the aged hardfacing coatings were more prone to pitting in pitting corrosion tests compared with the as-deposited hardfacing coatings. In addition to additive methods, cladding materials have also been extensively studied [16,17,18,19]. These studies have produced certain effects on the improvement in service life.

However, there are two shortcomings of traditional remanufacturing technology. The first is that the equal life design cannot be realized in different regions of a roller. The working conditions at different positions of a roller greatly vary in service, and it is difficult for one kind of material to meet different requirements for material performance under different working conditions. The second is that welding defects, such as thick columnar crystal structure, impurities, and shrinkage porosity, easily form in the weld pass. Meanwhile, the high-melting-point alloy elements first precipitate in the pool wall, resulting in uneven distribution of elements in the pool wall and weld center, which leads to regional segregation. Because of the isolation effect of coarse columnar crystals on regional segregation elements, it is difficult to eliminate regional segregation by heat treatment. The regional segregation, the as-cast structure and the residual tensile stress lead to stress corrosion spalling of the roller during service, and the weld bead trace greatly limits the improvement in the service life of the roller.

In order to solve these problems, which are encountered in traditional processes, a new remanufacturing process of rollers with a welding-hammering hybrid function is proposed and corresponding equipment was developed. The low-frequency hammering during welding can break columnar crystals, eliminate as-cast defects and reduce residual tensile stress. In addition, the roller was designed as a three-layer structure composed of a base layer, transition layer, and strengthened layer to improve the service life and reduce the repair costs. Through the gradient structure design of the roller and the welding-hammering hybrid process, the service life of the roller could be significantly improved while the repair costs could be reduced.

## 2. Failure Analysis of Continuous Casting Rollers

**Figure 1:**
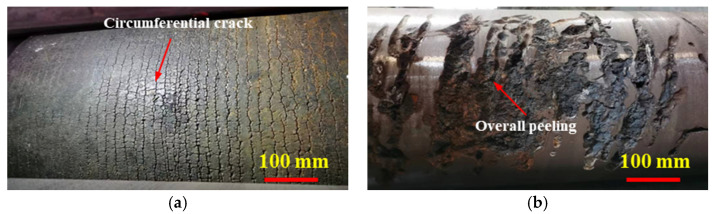
Two main failure modes of continuous casting rollers: (**a**) fatigue crack; (**b**) overall peeling.

Continuous casting rollers are used in high-temperature, high-stress, and highly corrosive environments. Therefore, the service life of a continuous casting roller is very short. Severe deformation occurs by particle separation due to friction and stress corrosion, leading to overall peeling and surface cracks (Figure 1) [20].

**Figure 2:**
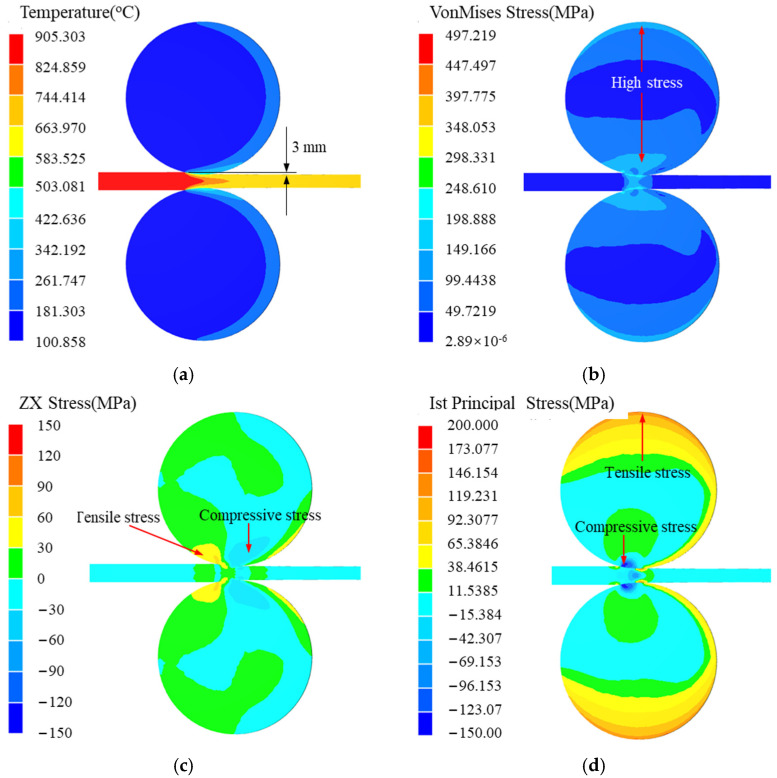
Simulation results of a roller service process: (**a**) temperature; (**b**) equivalent stress; (**c**) shear stress in rolling direction; (**d**) first principal stress.

The roller repaired by submerged arc welding mainly produces surface crack failure (Figure 1a), and overall peeling may occur in newly made rollers (Figure 1b). The roller surface is subjected to cyclic stress in a corrosive environment [21,22]. The simulation results of the roller service process are shown in Figure 2. The initial temperatures of the blank and the roller were set to 1000 °C and 100 °C, respectively. The materials of the roller and the blank were set to Cr5 and 45 steel, respectively. The simulation software was Forge NxT 1.0 which developed by TRANSVALOR S.A. Other simulation parameters are listed in Table 1.

As shown in Figure 2a, the roller surface temperature first increases, then decreases, and repeats continuously. In addition, the thickness of the high-temperature region on the roller surface is very small compared with the diameter of the roller. The equivalent stress on the upper and lower surfaces of the roller is high because the sheet metal exerts a reaction force on the roller, and then the upper surface of the roller is subjected to tensile stress. The shear stresses on the left and right of the contact surface between the roller and the blank are tension and compression, respectively (Figure 2c). The alternation of tension and compression shear stress occurs when the roller surface contacts the sheet metal, which leads to shear-fatigue and material falling off. The maximum principal stresses on the upper and lower surfaces of the roller are tensile and compressive stress, respectively (Figure 2d). This cyclic tension–compression normal stress leads to the fatigue failure of the materials, and then produces surface fatigue cracks. Therefore, there are cyclic tension–compression shear stress, cyclic tension–compression normal stress, thermal cycle, and other factors when the roller is in service. The service life of the roller can be very low when problems, such as segregation, as-cast defects, and mismatch of welding materials, exist in the weld.

## 3. Remanufacturing Process Design

### 3.1. Gradient Structure Design

According to previous analyses, the thickness of the high-temperature region on the roller surface is very small compared with the diameter of the roller. Materials with specific strengths at different temperatures are required for different regions. Therefore, the roller was divided into three layers along the radial direction, in which the roller surface has better high-temperature wear resistance and the middle layer has higher strength as supporting structure.

**Figure 3:**
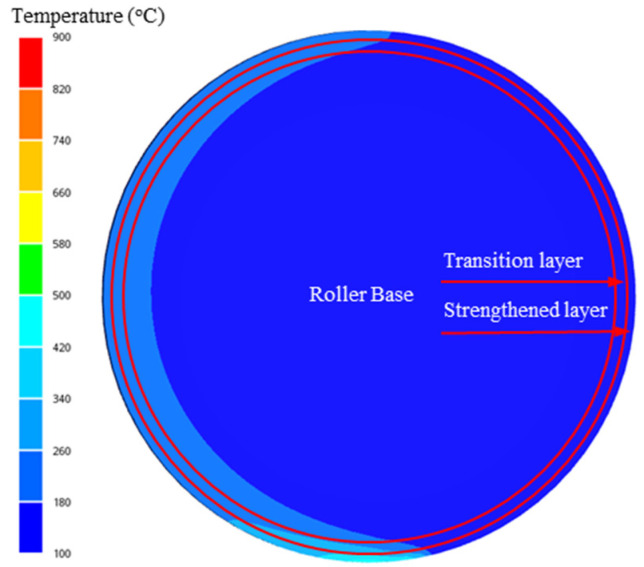
Three-layer gradient structure design of a continuous casting roller.

As shown in Figure 3, according to the temperature field simulation results, the thickness of the strengthened layer was designed as 3~5 mm. The thickness of the transition layer is determined by the crack depth before failure.

### 3.2. Materials Design

The strengthened layer of the roller needs high-temperature wear resistance, which is usually found in high-nickel and high-cobalt alloy materials [23,24]. A transition layer was used between the roller base and the strengthened layer, and an Fe-based alloy material (JX-401) was used to provide good support strength.

As shown in Table 2, a self-designed material, JX-422, was used as the strengthened layer, and JX-401 was used as the transition layer.

### 3.3. Wear Test

**Figure 4:**
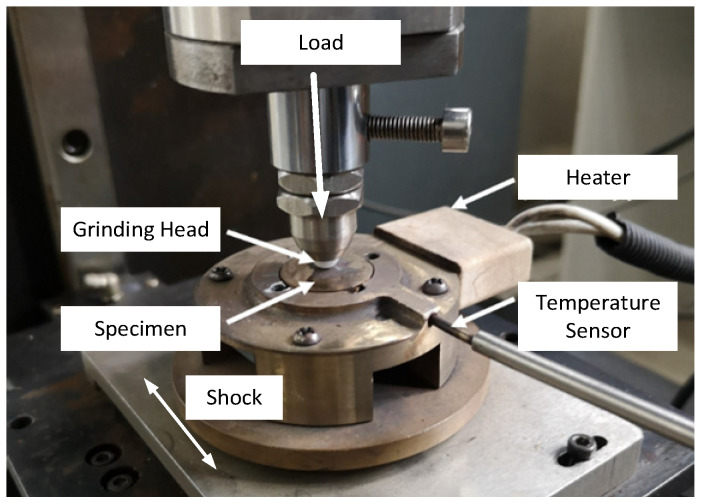
Wear resistance test equipment.

As shown in Figure 4, the experimental equipment was a ball disk high-speed linear reciprocating friction and wear tester MDW-02. The ball disk high-speed linear reciprocating friction and wear tester were mainly composed of a linear reciprocating mechanism, lifting mechanism, grinding head and its installation mechanism, sample stage, loading force sensor, friction sensor, heating rod, and temperature thermocouple, which could be used to study various metal and nonmetallic materials or their coatings at different temperatures, pressures, friction, and wear properties at sliding speed.

According to the clamping requirements of the sample stage, the size was Φ 20 mm. To avoid the influence of sample surface roughness and upper and lower layers of the sample on the high-temperature wear test, the sample surface was polished with 1200 # abrasive paper before the test. In order to avoid the influence of impurities and oil stains on the surface of the sample on the test results, before and after the high-temperature wear test, the sample was cleaned with an ultrasonic cleaner for 5 min and then cleaned with alcohol and blown dry. The detailed parameters of the wear test are shown in Table 3.

The wear experiments were performed to test the abrasion resistance of the materials. The wear volume under different wear times was measured at 200 °C and 500 °C.

**Figure 5:**
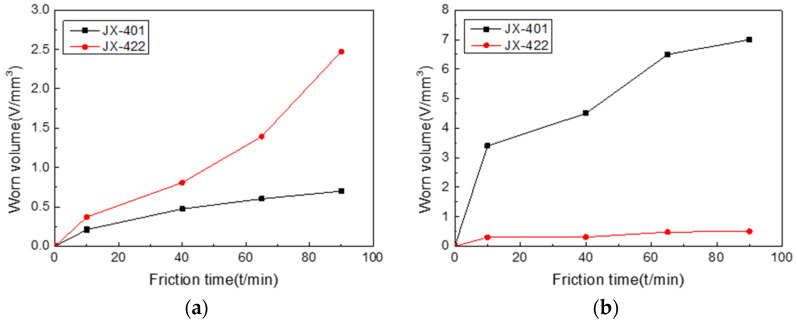
Wear volume of JX-422 and JX-401 at (**a**) 200 °C and (**b**) 500 °C.

**Figure 6:**
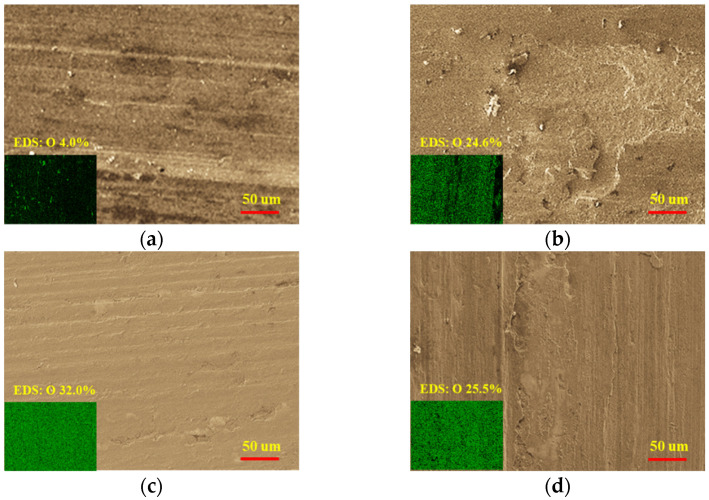
The wear surface morphology of two materials at different temperatures: (**a**) 200 °C, JX-401; (**b**) 200 °C, JX-422; (**c**) 500 °C, JX-401; (**d**) 500 °C, JX-422.

The wear test results (Figure 5) showed that the wear resistance of the Fe-based material (JX-401) was better than that of high-nickel alloy material (JX-422) at low temperatures. However, at high temperatures, the wear resistance of JX-422 was much better than that of JX-401. The wear resistance of JX-401 significantly decreased in high-temperature environments. The wear surface morphology of two materials at different temperatures is shown in Figure 6.

At low temperature, the wear mechanism of JX-401 was abrasive wear, and the wear mechanism of JX-422 was adhesive wear and slight oxidation wear. At high temperature, the wear mechanism of JX-401 was mainly oxidation wear and abrasive wear, and the wear mechanism of JX-422 as mainly oxidation wear and adhesive wear, accompanied by slight fatigue wear.

## 4. Welding-Hammering Hybrid Process and Application

### 4.1. Repair Equipment with Welding-Hammering Hybrid Function

**Figure 7:**
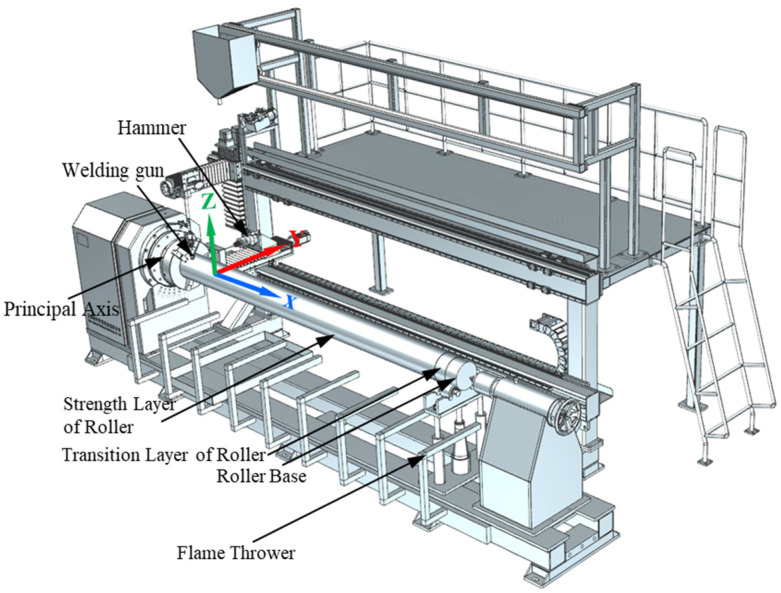
Repair equipment of roller with welding-hammering hybrid process.

Novel equipment that can conduct continuous welding-hammering hybrid remanufacturing was developed (as shown in Figure 7). The welding torch and hammer are set in vertical and horizontal directions, respectively. The z-direction movement of the welding torch and y-direction movement of the hammer are controlled by servo. In this way, the welding torch and hammer can move in the radial direction as well as in the axial direction. Through movement in these two directions and the rotation of the principal axis, a roller with any complex cross section can be filled and covered by irregular spiral lines, and thus be repaired by the welding-hammering hybrid process. The movements of the welding torch and hammer in the x direction do not share the slide track (the drive signal is shared). In this way, the position of welding and hammering in the x direction can be accurately matched, and the influence of hammer vibration on welding accuracy can be avoided.

As shown in Figure 7, a heating system that can control the roller temperature during the repair process was designed, and the flame thrower is controlled by a proportion integration differentiation (PID) controller. On one hand, a higher heating temperature is conducive to the elimination of the residual stress and cracks in the roller repair process. On the other hand, lower heating temperatures can avoid the microstructural changes and reduction in the mechanical properties of the roller base. Considering the above two aspects, the roller is heated to 500~600 °C during the repair process. Gas heating used in this newly developed equipment has many advantages such as fast heating speed, low energy consumption, low cost, and easy control, which can meet the large-scale needs of industry. One end of the clamping device is an adjustable three-jaw chuck; the other end is an adjustable thimble device; the bottom is a support bearing. In this way, rollers with different diameters can be fixed with this repair equipment.

### 4.2. Welding Process Parameters Analysis

**Figure 8:**
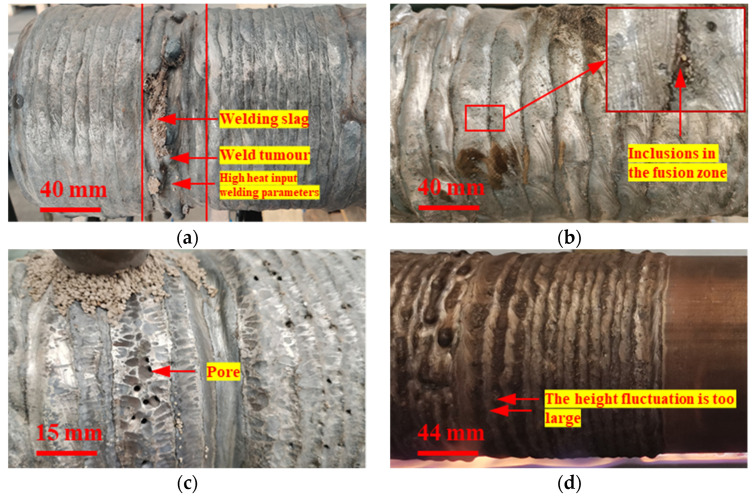
Common defects in roller repair: (**a**) weld tumor defect; (**b**) inclusion defect; (**c**) pore defect; (**d**) fluctuation defect.

A good welding process can not only meet the requirements of high-efficiency welding, but also can ensure welding quality. However, it is difficult to obtain the optimized welding parameters for small-diameter rollers. Figure 8 shows the welding defects, such as weld tumors, inclusions in the fusion zone, pores, fluctuation, and welding slags, which are caused by inappropriate welding process parameters. As shown in Figure 8a, excessive heat input leads to serious metal flow, and weld tumors form when the roller diameter is fixed, which is more serious for small-diameter rollers. However, a low heat input will reduce the repair efficiency. The actual welding process needs to balance the repair efficiency and metal flow according to different roller diameters.

As shown in Figure 8b, an improper linear energy density and overlap ratio cause the welding slag and metal oxide to be mixed between two adjacent welds. When the line energy density is small and the overlap rate is too large, a molten pool is formed on the surface of the previous weld, and the roller base cannot be fully melted. These conditions lead to welding slag, metal oxidation, and impurities at the edge of the molten pool, which cannot quickly rise to the surface, allowing inclusions to form. As shown in Figure 8c, if the water in the welding flux and welding wire, including free water and crystal water, is not completely removed, pores easily form in the weld. Therefore, the welding flux and welding wire (flux-cored wire) must be baked before welding. The composition of the welding flux is shown in Table 4. The welding flux should be kept at a temperature of 300~350 °C for two hours to remove the free and crystal water. As shown in Figure 8d, when a single weld is high and narrow, the flatness of the repaired surface is poor. Usually, a small voltage forms a narrow and high weld when other parameters are constant. After many experiments, the optimized process parameters for small-diameter rollers (less than 170 mm) were as follows: voltage 25 V, current 200 A, rotation speed 350 mm/min, wire extension 10 mm, eccentricity 8.5 mm, translation speed 5.24 mm/min, and the roller’s preheating temperature 550 °C.

### 4.3. Welding-Hammering Hybrid Process

**Figure 9:**
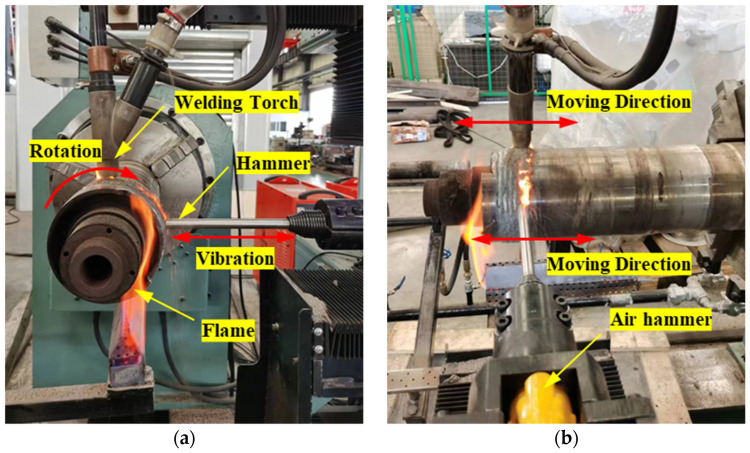
The repair site of a roller with welding-hammering hybrid process: (**a**) side view; (**b**) front view.

To obtain a smoother surface, improve the mechanical properties, and reduce the welding residual stress [25] of rollers after repairing, a welding-hammering hybrid process was designed. As shown in Figure 9, welding and hammering are carried out at the same time, and the weld metal is continuously rotated to the horizontal position for hammering. It is worth noting that the weld metal is still red when hammering, as shown in Figure 9b, and the hammering effect is best at this time. The best hammering temperature is usually slightly lower than the eutectoid-transformation temperature, which varies for different materials. The hammering temperature is usually 700~800 °C for steel. At this temperature, the weld metal is prone to deformation, and phase transformation does not occur after hammering. The compression deformation caused by hammering is greater than the volume shrinkage caused by metal cooling, which makes the residual tensile stress of the weld forcibly corrected to residual compressive stress. In addition, the microstructure of the weld material is refined due to the large hammering deformation at this temperature.

**Figure 10:**
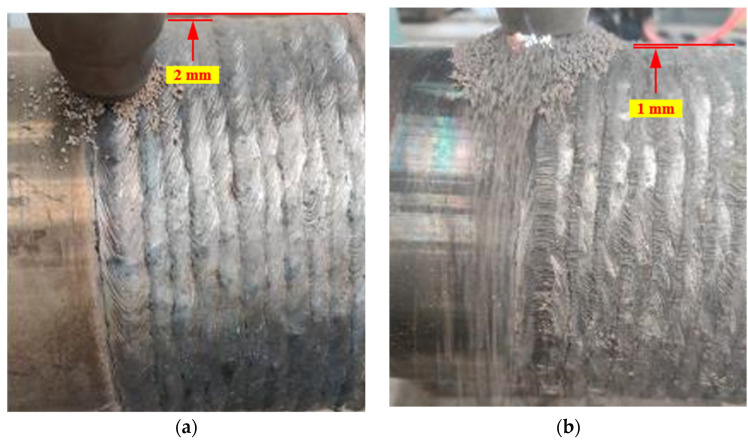
Surface morphology (**a**) without hammering and (**b**) with hammering under the optimized welding-process parameters.

The surface morphologies without and with a hammering process under the optimized welding process parameters are shown in Figure 10. As shown in Figure 10a, there are no macroscopic welding defects on the repaired surface and the repair quality is much better than that in Figure 7 under optimized welding-process parameters.

As shown in Figure 10b, the protruding metal on the weld surface is forcibly flattened by the hammering process, and the distance between the lowest and the highest points of the roller surface is reduced from 2 mm to 1 mm. The surface flatness is significantly improved after hammering, which can greatly reduce the machining allowance, thereby saving welding materials. In addition, the residual tension stress in welds can also be eliminated by hammering.

### 4.4. Application in Continuous Casting Roller Repair

**Figure 11:**
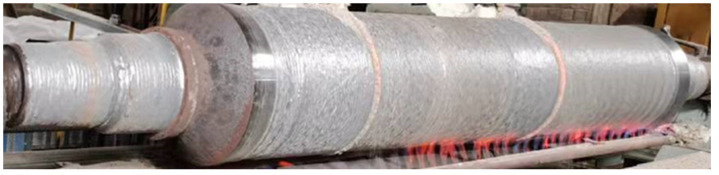
Roller repaired by the welding-hammering hybrid process.

Based on the analyses above, the process route of roller repair is as follows: removing the invalid material of the roller → preheating the roller to 550 °C → preheating the welding flux and welding wire → setting the welding process parameters and updating coordinate system → repairing the roller using welding-hammering hybrid process → heat treatment → machining → nondestructive testing. As shown in Figure 11, a continuous casting roller was repaired by the welding-hammering hybrid process with gradient structure.

**Figure 12:**
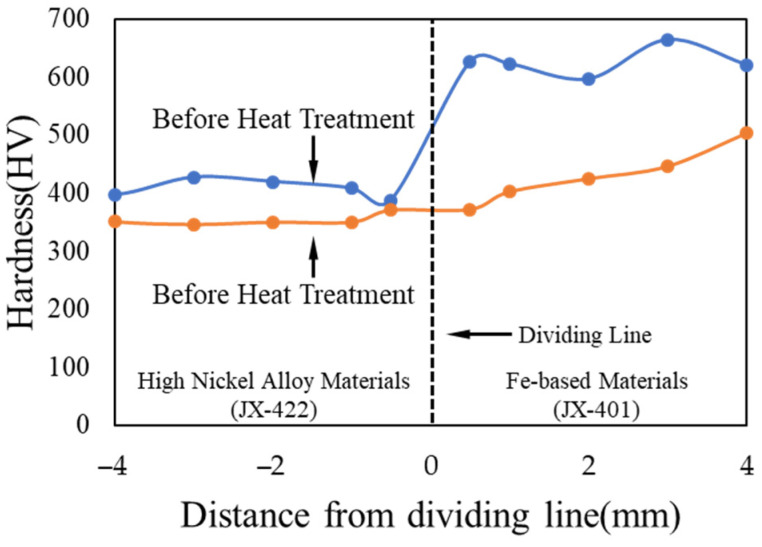
Hardness distribution in the direction of coating thickness.

The hardness distribution in the direction of coating thickness is shown in Figure 12. The hardness of high-nickel alloy materials and Fe-based materials is shown in Figure 11. The hardness of the high-nickel alloy is lower than Fe-based materials at low temperature. However, the wear resistance of high-nickel alloy is higher than that of Fe-based materials at high temperature.

**Figure 13:**
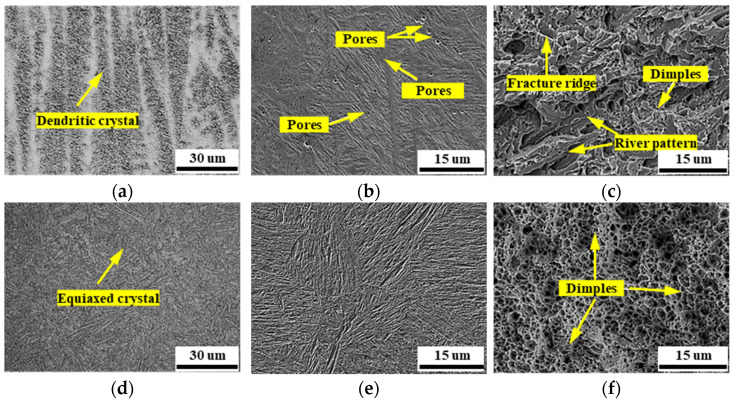
The micrographs of the welds with and without hammering: (**a**) microstructure of nonhammered specimen; (**b**) scanning electron microscope (SEM) of nonhammered specimen; (**c**) fracture morphology of nonhammered specimen; (**d**) microstructure of hammered specimen; (**e**) scanning electron microscope (SEM) of hammered specimen; (**f**) fracture morphology of hammered specimen.

To analyze the microstructure differences, the hammered and nonhammered repaired rollers were sampled, as shown in Figure 13.

As shown in Figure 13a,d, the microstructures of the nonhammered and hammered specimens presented a dendritic and equiaxed structure, respectively. The dendritic crystals can be defragmented by hammering, and then equiaxed crystals form. Compared with Figure 13e, the micropores existing in Figure 13b were eliminated by the welding-hammering hybrid process. Meanwhile, the fracture morphology changes from quasi-cleavage fracture characteristics in Figure 13c to ductile fracture characteristics in Figure 13f. This shows that the hammering process improves the toughness of the material. The microscopic analysis of the specimens shows that the welding-hammering hybrid process can refine the grain, eliminate welding defects, and improve the toughness of the material. Compared with traditional remanufacturing processes without hammering, the welding-hammering hybrid process produces a forged structure instead of an as-cast structure. The temperature of the weld metal is slightly lower than the solid’s temperature during hammering. Therefore, the weld grains are defragmented by the impact of the hammer. In addition, the pores are flattened by the impact of the hammer, and the internal air overflowed. The pores are largely eliminated after the second layer of metal cladding. Because the hammer impact causes plastic deformation of the metal, its grains can be refined and the as-cast defects can be largely eliminated so that the metal’s plasticity can be significantly improved.

The service status of two types of rollers is listed in Table 5. The service life of the newly manufactured roller is about 6 months, which is close to that of the repaired roller using traditional methods (without hammering and gradient structure). Under the same service conditions, the service life of the roller repaired by the welding-hammering hybrid process with gradient structure is about 12 months, which extends its life by about 100%.

## 5. Conclusions

(1) The failure modes of continuous steel casting rollers were studied, and the general approximate state of stress was specified by numerical simulation. The results showed that cyclic tension–compression shear stress, cyclic tension–compression normal stress, thermal cycle, and a highly corrosive environment cause the fatigue cracking and overall peeling of the roller surface.
(2) According to the simulation results of a roller under service conditions, a three-layer gradient structure composed of a base layer, transition layer, and strengthened layer was proposed. The strengthened layer of the roller needs high-temperature wear resistance, which is usually found in high-nickel and high-cobalt alloy materials. Then, weld materials JX-401, containing large amounts of Ni and Cr elements, and JX-422, containing large amounts of Cr element, were designed for the strengthened layer and transition layer, respectively. At high temperature, the wear mechanisms of JX-401 are mainly oxidation wear and abrasive wear, and the wear mechanisms of JX-422 are mainly oxidation wear and adhesive wear, accompanied by slight fatigue wear. The wear resistance of JX-422 is significantly higher than that of JX-401 at high temperature.
(3) The new welding-hammering hybrid process was applied to repair a continuous casting roller. The microscopic analysis of the specimens showed that the welding-hammering hybrid process can defragment the crystal, eliminate the welding pores, and improve the toughness of the material. The repair process without hammering can only produce an as-cast structure, but the welding-hammering hybrid process can produce a forged structure. The new welding-hammering hybrid process can significantly improve the service life of a repaired roller by about 100% compared with traditional methods.

## Figures and Tables

**Table 1:** Finite element simulation parameters in the simulation process.

Roller Diameter(mm)	Initial Temperature (°C)		Number of Mesh		Friction Model		Heat Transfer Coefficient (W/m^2^K)
	Blank	Roller	Blank	Roller	Coulomb	Shear	
170	1000	100	200,000	10,000	0.15	0.3	20,000

**Table 2:** Composition of welding materials in different layers (wt.%).

Material	C	Si	Mn	Cr	Mo	W	V	Ni	Co	Fe
JX-401 (Transition Layer)	0.06	0.7	1.20	17.0	2.42	1.79	0.35	-	-	Bal.
JX-422 (Strengthened Layer)	0.03	2.0	2.00	21.0	3.50	-	0.35	11.00	0.35	Bal.
Cr5 (Roller Base)	0.52	0.46	0.55	5.10	0.60	-	0.11	0.43	-	Bal.

**Table 3:** Parameters of wear test.

Parameter	Value
Load (*F*/N)	200
Temperature (*T*/°C)	200, 500
Moving Frequency (*f*/Hz)	4
Moving Stroke (*L*/mm)	8
Wear Time (*t*/min)	10, 40, 65, 90

**Table 4:** The composition of welding flux (wt.%).

SiO_2_ + TiO_2_	MnO + A1_2_O_3_	CaO + MgO	CaF_2_	S	P	H_2_O	Impurity
19.7	24.2	31.7	20.5	0.013	0.017	0.02	0.02

**Table 5:** Service status of rollers.

Service Conditions of Roller				New Roller Service Results		Repaired Roller Service Results (with Hammering)	
Roller Surface Temperature (°C)	Roller Diameter (mm)	Rotation Speed (r/min)	Rolling Reduction (mm)	Rolling Capacity (T)	Service Time (Month)	Rolling Capacity (T)	Service Time (Month)
500~600	170	700	10	150,000	6	300,000	12

## Data Availability

All data generated or analyzed during this study are included in this published article.
